# Progress on the Experimental Research of Sciatic Nerve Injury with Acupuncture

**DOI:** 10.1155/2021/1401756

**Published:** 2021-12-23

**Authors:** Hui Wang, Jingjing Cui, Shitong Zhao, Dongsheng Xu, Shuang Wu, Wanzhu Bai, Jia Wang

**Affiliations:** ^1^Institute of Acupuncture and Moxibustion, China Academy of Chinese Medical Sciences, Beijing 100700, China; ^2^Center for Experimental Medicine, The First Affiliated Hospital of Nanchang University, Nanchang, Jiangxi 330006, China; ^3^Department of Traditional Chinese Medicine, Beijing Chaoyang Hospital, Capital Medical University, Beijing, China

## Abstract

**Objective:**

To collect and summarize relevant literatures on the experimental researches of sciatic nerve injury (SNI) with acupuncture during the last decade providing a guideline for effectively treating SNI with acupuncture in the future.

**Methods:**

The Chinese and English databases including China National Knowledge Infrastructure (CNKI), Wanfang Data Knowledge Service Platform (WanFang Data), VIP Information Chinese Journal Service Platform (VIP Date), and PubMed were searched from 2009 to 2020 with keywords of “acupuncture and moxibustion OR acupuncture OR electroacupuncture OR scalp acupuncture OR wrist-ankle acupuncture OR acupoint injection OR ear acupuncture” AND “sciatic nerve OR sciatic nerve injury OR sciatic injury OR SNI.” The collected data were mainly evaluated in the items of animal model of SNI, type of interventions, selection of acupuncture points (acupoints), course of treatment and its frequency, and approaches of assessment.

**Results:**

A total of 89 studies were included in this analysis. Among them, the most commonly used animal models of SNI were produced by the clamp or transverse injury in the rats; the most frequently used intervention was electroacupuncture with dilatational wave of 2/100 Hz; the frequency of acupuncture was mainly performed once per day lasting for more than 2 weeks; the mainly selected acupoints were Huantiao (GB30), Zusanli (ST36), and Yanglingquan (GB34); and the approaches of assessment were contained with behavioral, functional, morphological, histological, cellular, and molecular measurements.

**Conclusion:**

The results indicated that the experimental researches of SNI with acupuncture has made marked progress in recent years, which may provide important clues for further investigating the underlying mechanisms of acupuncture for the treatment of SNI in the future.

## 1. Introduction

Sciatic nerve injury (SNI), as a typical type of peripheral nerve injury, is a common disorder to meet the acupuncture treatment [1–4]. Usually, SNI might be caused by incidents such as trauma, crush injury, sharp instrument attacking, drug injection, pelvic fracture, and hip dislocation, manifesting with sensory dysfunction such as pain, numbness, and loss of sensation, as well as locomotor dysfunction such as muscular atrophy, muscular tone decreasing, and limb paralysis [1, 5–7]. Although SNI is not lethal, it can cause long-term functional deficits in patients [8, 9].

Up to now, in order to treat SNI, many efforts have been made in clinical practices, including the transplantation of neural tissues and stem cells, the administration of neurotrophic medicine and nerve growth factor, as well as physical and laser therapies [10, 11]. Although all of them had certain curative effects on the SNI, considering their side effects and relatively high costs, some of the patients also accepted acupuncture as a complementary or alternative choice and obtained satisfied effects from treatment [12–14]. However, the underlying mechanism of acupuncture for meliorating SNI still remains unclear.

In order to tackle this problem, a large number of experimental researches on SNI with acupuncture has been carried out and reported separately in the last decade [15–17]. In this study, we collected the relevant literatures in this field from the Chinese and English databases with correlated keywords and evaluated them in the items of the animal model of SNI, type of interventions, selection of acupuncture points (acupoints), course of treatment and its frequency, and approaches of assessment, respectively. By analyzing these data, we expect to summarize the progress on the experimental research of SNI with acupuncture and provide a clue for further investigating its underlying mechanism associated with the clinical application.

## 2. Materials and Methods

### 2.1. Criteria for Study Inclusion and Exclusion

1. Type of studies: the original studies related to the experimental research of acupuncture for SNI were included. However, the clinical trials, experience introductions, reviews, and other relevant literatures would be excluded.
2. Type of research object: rat, rabbit, mouse, cat, monkey, dog, etc., no limitation on animal species and gender.
3. Type of intervention: any type of acupuncture was included, including acupuncture, electroacupuncture, scalp acupuncture, ear acupuncture, wrist-ankle acupuncture, and acupoint injections.
4. Types of outcomes: the animal model of SNI, type of interventions, selection of acupoints, course of treatment and its frequency, and approaches of assessment were mainly analyzed.

### 2.2. Search Strategy

Taking “acupuncture and moxibustion OR acupuncture OR electroacupuncture OR scalp acupuncture OR wrist-ankle acupuncture OR ear acupuncture OR acupoint injection” AND “sciatic nerve OR sciatic nerve injury OR sciatic injury OR SNI” as the keywords searched for the literature related to acupuncture intervention for SNI on the Chinese and English databases, including China National Knowledge Infrastructure (CNKI, 2009–2020), Wanfang Data Knowledge Service Platform (WanFang Data, 2009–2020), VIP Information Chinese Journal Service Platform (VIP Date, 2009–2020), and PubMed (2009–2020).

### 2.3. Data Extraction and Analysis

Two reviewers (Hui Wang and Jia Wang) independently used a predesigned data extraction form for rigorous data collection, including general information such as authors of studies, year of publication, and animal model of SNI, as well as treatment information including type of intervention, selection of acupoints, course of treatment and its frequency, and approaches of assessment. Finally, it would be systematically summarized and analyzed.

## 3. Results

### 3.1. Study Selection

Through electronic searching, 10795, 13746, and 8562 records in Chinese were identified from CNKI, Wanfang, and VIP Date databases, respectively, and 166 records in English were found from PubMed. By eliminating studies that were irrelevant, repetitive, and unable to obtain the full text, there were totally 89 studies included in accordance with the inclusion and exclusion criteria. Among these, there were 83 studies published in Chinese and 6 studies in English.

### 3.2. Characteristics of Included Studies

#### 3.2.1. Annual Publication

**Figure 1:**
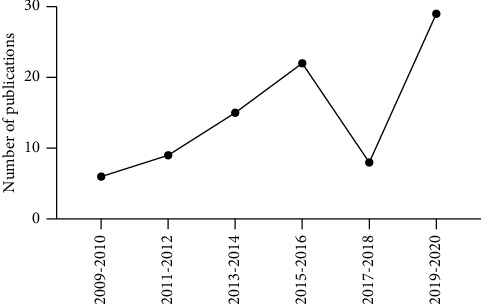
Publication status of experimental research of SNI with acupuncture during 2009–2020.

During the past twelve years (2009–2020), there has been an overall upward trend in the number of annual publications on acupuncture treatment for SNI. Counting by the number of publications in a two-year interval, it was observed that the highest number of publications appeared in 2019–2020 with 29 studies, followed by 2015–2016 with 22 studies (Figure 1).

#### 3.2.2. Animal Species

**Figure 2:**
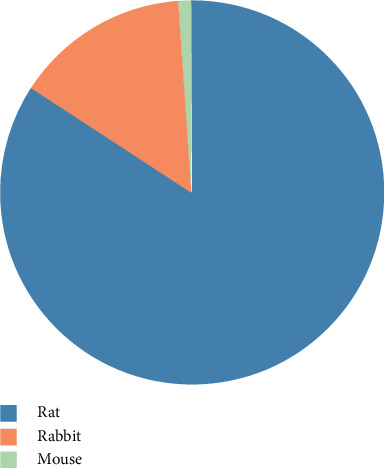
Animal application status in experimental research of SNI with acupuncture.

In the 89 included studies, the animals used for preparing the SNI model included rats, mice, and rabbits. Among them, the rat was by far the most commonly employed animal model in SNI experimental research, with the rabbit being the second most popular model, accounting for 84.3% (75/89) and 14.6% (13/89), respectively. There was only one study that applied to the mouse (Figure 2).

#### 3.2.3. Animal Model of SNI

By summarizing the included studies, the animal models of SNI were established by many methods, including transverse, clamp, ligation, or injection injury to sciatic nerve [18–21]. Among them, the model with clamp injury was the most frequently used, and the model with transverse injury was the second most applied, with 61 (61/89, 68.5%) and 19 studies (19/89, 21.3%), respectively.

### 3.3. Application Characteristics of Acupuncture on SNI

#### 3.3.1. Type of Interventions

**Figure 3:**
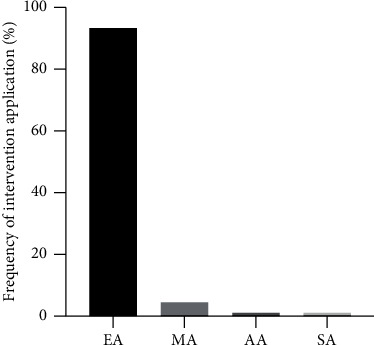
The intervention application status in experimental research of SNI with acupuncture. Note. EA: electroacupuncture; MA: manual acupuncture; AA: ankle acupuncture; SA: scalp acupuncture.

Among the 89 included studies, the types of interventions mainly included electroacupuncture, manual acupuncture, ankle acupuncture, and scalp acupuncture, accounting for 93.3% (83/89), 4.5% (4/89), 1.1% (1/89), and 1.1% (1/89), respectively (Figure 3). In some of the included studies, multiple interventions of acupuncture were simultaneously used.

In the 83 studies of electroacupuncture, besides 7 studies that did not clearly indicate the stimulated parameters, the other 76 studies marked the definite stimulated styles, in which the waveforms of electroacupuncture were used orderly with dilatational wave (64.5%, 49/76), sparse wave (19.7%, 15/76), continuous wave (11.8%, 9/76), and intermittent wave (3.9%, 3/76). The frequencies of electroacupuncture were applied orderly with 2/100 Hz (64.5%, 49/76), ＜10 Hz (19.7%, 15/76), 15 Hz (9.2%, 7/76), and 20 Hz (6.6%, 5/76), and the electric current and voltage were used to cause mild muscle tremors (84.2%, 64/76), 1.5 mA (3.9%, 3/76), 2 mA (3.9%, 3/76), 20 mV (3.9%, 3/76), and 1.5 V (3.9%, 3/76). In addition, the time of treatment was varied from 9 min to 30 min in 80 studies in order of 20 min (36.25%, 29/80), 10 min (31.25%, 25/80), 15 min (25.0%, 20/80), 30 min (3.75%, 3/80), and 9 min (3.75%, 3/80).

#### 3.3.2. Selection of Acupoints

**Figure 4:**
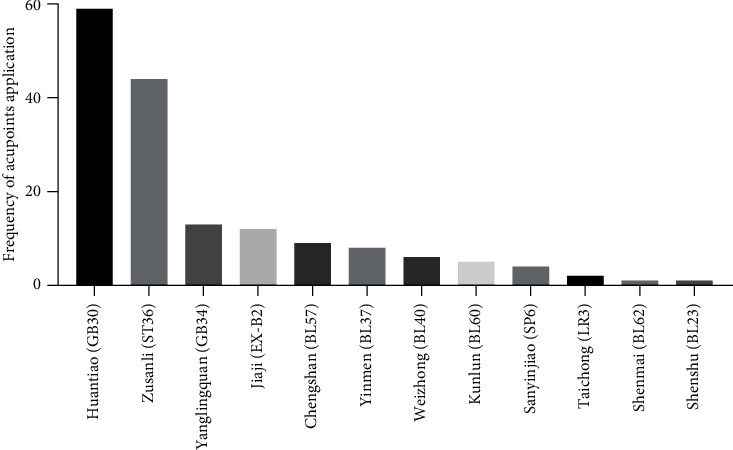
The acupoint selection in experimental research of SNI with acupuncture.

A total of 13 acupoints appeared in the 89 studies, in which Huantiao (GB30, 59/89, 66.3%) and Zusanli (ST36, 44/89, 49.4%) appeared most frequently, followed with Yanglingquan (GB34, 13/89, 14.6%) and Jiaji (EX-B2, 12/89, 13.5%) (Figure 4).

**Figure 5:**
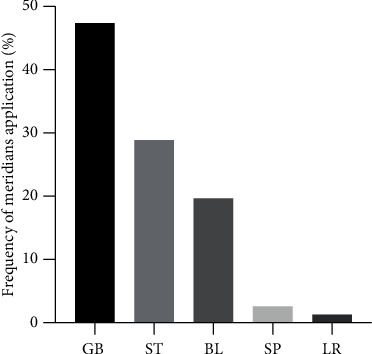
The meridians selection in experimental research of SNI with acupuncture. Note. GB: gallbladder meridian; ST: stomach meridian; BL: bladder meridian; SP: spleen meridian; LR: liver meridian.

Except Jiaji, the other acupoints mainly belong to the 5 meridians of the gallbladder (GB), stomach (ST), bladder (BL), spleen (SP), and liver (LR) meridians (Figure 5).

#### 3.3.3. Treatment Frequency and Course

In the included studies, only one study did not mention the frequency of acupuncture. Among the other 88 studies, most studies applied the frequency of once per day (86/88, 97.7%). Only 2 studies applied once in every 2 days (2/88, 2.3%).

Regarding the treatment course, many studies performed acupuncture for more than 2 weeks (56.2%, 50/89), while another 33 studies performed for 1–2 weeks (37.1%, 33/83), and 6 studies performed for less than 1 week (6.7%, 6/89).

### 3.4. Analysis Methods Related to SNI with Acupuncture

#### 3.4.1. Behavioral Measurement

Among the total included studies, 52 studies applied the behavioral measurements, including sciatic nerve function index (SFI), pain threshold, toe abduction reflex, and inclined pulling test. Among them, SFI was the most widely used (57.7%, 30/52), followed by the measurement of thermal pain threshold (30.8%, 16/52). In addition, 15.6% (5/52) and 1.9% (1/52) of studies applied the toe abduction reflex and inclined pulling test, respectively.

#### 3.4.2. Functional Assessment

There were 33 studies that applied the functional assessment, including nerve and muscle function assessments. Among them, 30 studies mainly measured the sciatic nerve function as well as sensory and motor nerve conduction velocity. Another 3 studies measured electromyography and muscle contractility.

#### 3.4.3. Morphological, Histological, Cellular, and Molecular Measurements

**Figure 6:**
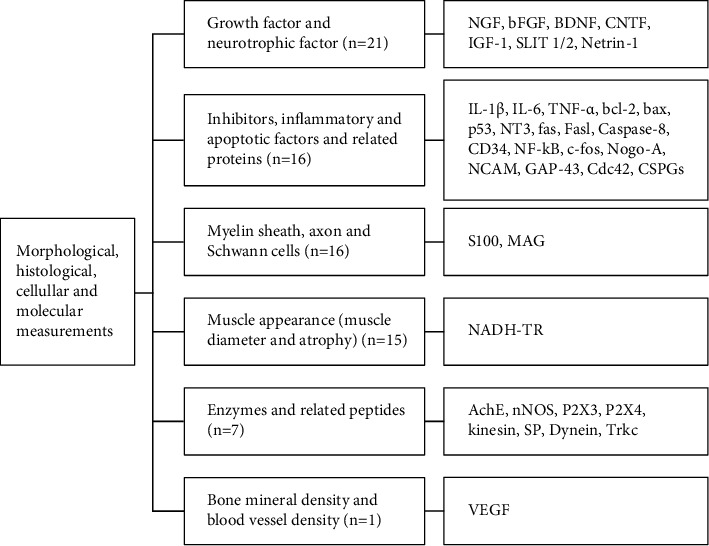
The detailed measurements and indicators in the experimental research of SNI with acupuncture. Note: AchE: acetylcholinesterase; bcl-2: b cell lymphoma-2; BDNF: brain-derived neurotrophic factor; bFGF: basic fibroblast growth factor; Cdc42: cell division cycle 42; CNTF: ciliary neurotrophic factor; CSPGs: chondroitin sulfate proteoglycans; GAP-43: growth-associated protein-43; IGF-1: insulin-like growth factor 1; IL-1*β*: interleukin-1 beta; IL-6: interleukin 6; MAG: myelin-associated glycoprotein; NADH-TR: nicotinomide adenine dinucleotide dehydrogenase-tetrazolium reductase; NCAM: neural cell adhesion molecule; NF-kB: nuclear factor kB; NGF: nerve growth factor; NT3: neurotrophin-3; nNOS: neuronal nitric oxide synthase; SLIT 1/2: slit guidance ligand 1/2; SP: substance P; TNF-*α*: tumor necrosis factor-*α*; Trkc: tropomyosin receptor kinase C; VEGF: vascular endothelial growth factor.

The approaches of assessments related to the effects and mechanisms of SNI with acupuncture mainly included morphological, histological, cellular, and molecular measurements. There were 58 studies that applied morphological and histological testing, and 44 studies used cellular and molecular testing. The spinal cord, dorsal root ganglia (DRG), sciatic nerve and its related muscles were the main tissues to be examined. The growth factor and neurotrophic factor were the most frequently tested indicators, with 21 studies applied. Besides, inhibitors inflammatory and apoptotic factors, and related proteins were commonly detected in 16 studies. Besides, there were 16 studies focused on the detection of the myelin sheath, axon, and Schwann cells of the sciatic nerve, and 15 studies focused on the muscle appearance (measurement of muscle diameter and atrophy). Additionally, there were 7 studies that paid attention to the testing of enzymes and related peptides, but only one study had done the observation on the bone mineral density and blood vessel density tests. The detailed description of the molecules or factors involved are displayed in Figure 6.

### 3.5. Observation on the Mechanism of SNI with Acupuncture

According to the summary of the research in the past twelve years, the current speculated relevant mechanisms of SNI with acupuncture may include the following: ① acupuncture could promote nerve regeneration and inhibit cell apoptosis by promoting the expression of nerve growth factor in SNI [22–24]; ② acupuncture could reduce the expression of inflammatory factors and increase the content of related proteins to promote the recovery of SNI [25, 26]; and ③ acupuncture could promote the proliferation of Schwann cells to the injured sciatic nerve to protect the nerve [27–29]. However, the research on the mechanism was mostly scattered, and they still could not represent the exact mechanism of acupuncture for SNI.

## 4. Discussion

In this study, the data about the experimental research of SNI with acupuncture has been collected from 2009 to 2020 and summarized in the items of animal model of SNI, type of interventions, selection of acupoints, course of treatment and its frequency, as well as approaches of assessment (Table 1). During the past twelve years, research in this field has made marked progress, step by step, to understand the underlying mechanisms of why acupuncture can play an effective role in alleviating SNI.

It is well known that, from bench to bed, experimental research is an indispensable way to promote the development of clinical progress. Although animal models with different types of SNI have been introduced to experimental research, such as transverse, ligation, clamp, traction, freezing, or drug injuries to the sciatic nerve, it is still difficult to select an appropriate one for mimicking the clinical patients. Among these models, the model with clamp injury is widely used to evaluate the effect of acupuncture on neural recovery [15, 17, 19, 30], and the model with transverse injury is mainly applied to assess the effect of acupuncture on neural regeneration [31]. Besides these two types, the model with ligation injury is also used; however, it seems difficult to control the tightness of the ligation in unity [20, 32]. For the model with traction injury, it is also difficult to control the elongation of the sciatic nerve with two forceps between both ends of the nerve [33]. Additionally, the model with freezing injury is also applied. The problem is that the cryocooling probe not only damages the nerve, but also involves its surrounding tissues [33]. Therefore, considering the advantages and disadvantages of these animal models, the model with clamp injury is more suitable for investigating the underlying mechanism of acupuncture for meliorating the SNI.

In terms of the interventions for the SNI, electroacupuncture was recommended according to the collected data, which is consistent with the situation in clinical treatment. The possible reason might be that the parameters of stimulation with electroacupuncture can be controlled as demands on its strength and frequency [34]. Indeed, numerous studies have demonstrated that electroacupuncture has a good analgesic effect on various kinds of painful diseases [35, 36]. Here, it should be noted that although the 2/100 Hz dilatational wave is an appropriate choice for the animal models [37, 38] since the body size is different between small animals and humans, whether this parameter is suitable for the patients remains unanswered.

For the selection of acupoints, it is clear that, besides Jiaji (EX-B2) on the back, the other twelve acupoints are located closely or along the pathway of the sciatic nerve including its main trunk and its branches of the tibial, common peroneal, and sural nerves [39]. Traditionally, these acupoints belong to the meridians of the gallbladder (GB), stomach (ST), bladder (BL), spleen (SP), and liver (LR), respectively, and are orderly arranged along the longitudinal axis of the hind limb [39]. Although GB30 and ST36 are used most frequently, most acupoints belong to the bladder meridian including BL23, BL37, BL40, BL57, BL60, and BL62. Coincidently, the course of the bladder meridian as the traditional description is similar to the pathway of the sciatic nerve [39]. According to the spatial correlation between these acupoints and the sciatic nerve, they are the adjacent points to the sciatic nerve or its branches. In the clinical treatment of SNI, these acupoints can be used individually or together.

Although acupuncture treatment has obtained satisfactory effects on patients with SNI, due to the limitation of clinical studies, these effects are mainly evaluated on the variation of clinical symptoms and are seldom involved in their underlying mechanism [12–14]. As a comparison, experimental research has its own advantages in obtaining much more biological information from the perspective of pathologic histology. According to our collected data, most studies on the SNI with acupuncture were concentrated on neural protection and neural regeneration by way of anti-inflammation and antiapoptosis [22–27]. These studies suggested that acupuncture could promote nerve regeneration, inhibit cell apoptosis, reduce the expression of inflammatory factors, improve the microenvironment of the injured area, and promote the proliferation of Schwann cells to repair the injured sciatic nerve [22–27]. However, there is still an absence of high-quality studies in this field. Therefore, the definite mechanism associated with acupuncture treatment on the SNI still needs further investigation in the future. Recently, there has been a new trend in SNI studies. Some researchers have paid attention to the SNI from the peripheral nervous system to the central nervous system, including the sensory input to the spinal dorsal horns and motor output from the spinal ventral horns [25, 26, 40]. It might be a new direction for acupuncture research on the SNI in the future.

## 5. Conclusion

In summary, the experimental research on the SNI with acupuncture has made marked progress in recent years, providing rich evidence for insight into the underlying mechanisms of acupuncture and how to alleviate the SNI. These experimental data are not only beneficial to future studies in this field but also for selecting appropriate acupoints and stimulating styles as clinical demands with SNI.

## Figures and Tables

**Table 1:** Summary table showing the application characteristics of acupuncture on SNI.

Treatment strategy	Selected acupoints	Treatment frequency	Treatment course	Stimulation parameters of EA				Analysis methods	Detailed molecules or factors
				Waveform	Frequency	Current or voltage	Time of treatment		
EA (93.3%) MA (4.5%) AA (1.1%) SA (1.1%)	Huantiao (GB30, 66.3%) Zusanli (ST36, 49.4%) Yanglingquan (GB34, 14.6%) Jiaji (EX-B2, 13.5%), Chengshan (BL57, 10.1%), Yinmen (BL37, 9.0%), Weizhong (BL40, 6.7%), Kunlun (BL60, 5.6%), Sanyinjiao (SP6, 4.5%), Taichong (LR3, 2.2%), Shenmai (BL62, 1.1%), and Shenshu (BL23, 1.1%)	Once per day (97.7%); once every 2 days (2.3%)	＞2 W (56.2%); 1–2 W (37.1%); ＜1 W (6.7%)	Dilatational wave (64.5%) Sparse wave (19.7%) Continuous wave (11.8%) Intermittent wave (3.9%)	2/100 Hz (64.5%); ＜10 Hz (19.7%); 15 Hz (9.2%); 20 Hz (6.6%)	Cause mild muscle tremor (84.2%); 1.5 mA (3.9%) 2 mA (3.9%); 20 mV (3.9%); 1.5 V (3.9%)	20 min (36.25%); 10 min (31.25%); 15 min (25.0%); 30 min (3.75%); 9 min (3.75%)	① Behavioral measurement (SFI, pain threshold, toe abduction reflex, and inclined pulling test); ② Functional assessment (sensory and motor nerve conduction velocity, electromyography, and muscle contractility); ③ Morphological, histological, cellular, and molecular measurements	① Growth factor and neurotrophic factors (NGF, bFGF, BDNF, CNTF, IGF-1, SLIT 1/2, Netrin-1) ② Inhibitors, inflammatory, and apoptotic factors and related proteins (IL-1*β*, IL-6, TNF-*α*, bcl-2, bax, p53, NT3, fas, fasL, caspase-8, CD34, NF-kB, c-fos, nogo-a, NCAM, GAP-43, Cdc42, and CSPGs) ③ Muscle appearance (NADH-TR) ④ Enzymes and related peptides (AchE, nNOS, P2X3, P2X4, kinesin, SP, dynein, andTrkc) ⑤ Myelin sheath, axon, and Schwann cells (S100, MAG) ⑥ Bone mineral density and blood vessel density (VEGF)

*Note.* EA: electroacupuncture; MA: manual acupuncture; AA: ankle acupuncture; SA: scalp acupuncture; W: week; AchE: acetylcholinesterase; bcl-2: b cell lymphoma-2; BDNF: brain-derived neurotrophic factor; bFGF: basic fibroblast growth factor; Cdc42: cell division cycle 42; CNTF: ciliary neurotrophic factor; CSPGs: chondroitin sulfate proteoglycans; GAP-43: growth-associated protein-43; IGF-1: insulin-like growth factor 1; IL-1*β*: interleukin-1 beta; IL-6: interleukin 6; MAG: myelin-associated glycoprotein; NADH-TR: nicotinomide adenine dinucleotide dehydrogenase-tetrazolium reductase; NCAM: neural cell adhesion molecule; NF-kB: nuclear factor kB; NGF: nerve growth factor; NT3: neurotrophin-3; nNOS: neuronal nitric oxide synthase; SLIT 1/2: slit guidance ligand 1/2; SP: substance P; TNF-*α*: tumor necrosis factor-*α*; Trkc: tropomyosin receptor kinase C; VEGF: vascular endothelial growth factor.

## Data Availability

The datasets used for the current study are available from the corresponding author upon request.
